# Frequency‐specific changes in the default mode network in patients with cingulate gyrus epilepsy

**DOI:** 10.1002/hbm.24956

**Published:** 2020-02-25

**Authors:** Xuerong Leng, Jing Xiang, Yingxue Yang, Tao Yu, Xiaohong Qi, Xiating Zhang, Siqi Wu, Yuping Wang

**Affiliations:** ^1^ Department of Pediatrics Xuanwu Hospital Capital Medical University Beijing China; ^2^ MEG Center, Division of Neurology Cincinnati Children's Hospital Medical Center Cincinnati Ohio; ^3^ Department of Neurology Xuanwu Hospital Capital Medical University Beijing China; ^4^ Beijing Key Laboratory of Neuromodulation Beijing China; ^5^ Center of Epilepsy Beijing Institute for Brain Disorders, Capital Medical University Beijing China; ^6^ Beijing Institute of Functional Neurosurgery Xuanwu Hospital, Capital Medical University Beijing China

**Keywords:** angular gyrus, cingulate gyrus epilepsy, default mode network, magnetoencephalography

## Abstract

To identify abnormal functional connectivity of the default mode network in cingulate gyrus epilepsy, which may yield new information about the default mode network and suggest a new cingulate gyrus epilepsy biomarker. Fifteen patients with cingulate gyrus epilepsy (mean age = 21 years) and 15 healthy controls (mean age = 24 years) were studied in the resting state using magnetoencephalography. Twelve brain areas of interest in the default mode network were extracted and investigated with multifrequency signals that included alpha (α, 8–13 Hz), beta (β, 14–30 Hz), and gamma (γ, 31–80 Hz) band oscillations. Patients with cingulate gyrus epilepsy had significantly greater connectivity in all three frequency bands (α, β, γ). A frequency‐specific elevation of functional connectivity was found in patients compared to controls. The greater functional connectivity in the γ band was significantly more prominent than that of the α and β bands. Patients with cingulate gyrus epilepsy and controls differed significantly in functional connectivity between the left angular gyrus and left posterior cingulate cortex in the α, β, and γ bands. The results of the node degree analysis were similar to those of the functional connectivity analysis. Our findings reveal for the first time that brain activity in the γ band may play a key role in the default mode network in cingulate gyrus epilepsy. Altered functional connectivity of the left angular gyrus and left posterior cingulate cortex may be a new biomarker for cingulate gyrus epilepsy.

**Abbreviations**
DMN: default mode network
MEG: magnetoencephalography
FC: functional connectivity

## 1 INTRODUCTION

The cingulate cortex includes Brodmann areas 23, 24, 25, 29, 30, 31, 32, and 33, and is a complex central structure in the human brain (Brodmann, 1909). It is related to diverse functions (visuospatial, memory functions, emotion, cognition, premotor activity, behavior monitoring, error detection, and adaptive decision making) (Walton, Croxson, Behrens, Kennerley, & Rushworth, 2007). The cingulate cortex is believed to play an important role in the propagation of epileptic discharges on account of its diffuse connections (Alkawadri, Mickey, Madden, & Van Ness, 2011; Devinsky, Morrell, & Vogt, 1995). A recent study indicated that patients with idiopathic generalized epilepsy have structural abnormalities in the cingulate gyrus (Braga et al., 2015).

The International League Against Epilepsy (ILAE; Commission on Classification and Terminology of the International League Against Epilepsy., 1989) classified cingulate gyrus epilepsy as a type of frontal lobe epilepsy in the proposed classification of epilepsy and epileptic syndromes in 1989. ILAE described cingulate gyrus epilepsy as consisting of complex partial seizures with complex motor gestural automatisms at onset, autonomic signs, and changes in mood and affect. However, cingulate gyrus epilepsy is disputed because it may have diverse clinical features or features that may overlap with other frontal lobe epilepsy syndromes. Due to its anatomical location, recording epileptic discharges directly from the cingulate gyrus using scalp electrodes is difficult. Therefore, locating the epileptogenic zone in the cingulate gyrus is difficult; thus, studies of clinical symptoms and electrophysiology in patients with cingulate gyrus epilepsy are rarely conducted or reported.

The default mode network (DMN) was first proposed by Raichle and colleagues and describes the areas of the brain that are more active during a state of alert, awake but not actively goal‐directed behavior (Raichle et al., 2001). Many anatomically separated cortical regions compose the DMN, which contains the posterior cingulate cortex (PCC), precuneus (PCUN), inferior parietal cortex (IPC) (mainly angular gyrus; AG), medial temporal (MT) lobes, medial prefrontal cortex (MPFC), and anterior cingulate cortex (ACC; Greicius, Krasnow, Reiss, & Menon, 2003; Greicius & Menon, 2004; Raichle et al., 2001). The PCC is suggested as the only node in the DMN that directly interacts with almost all other nodes according to Fransson and Marrelec (2008). Various brain regions that comprise the DMN are involved in the integration of autobiographic, self‐monitoring, and social cognitive functions (Spreng, Mar, & Kim, 2009). However, the precise functions collectively served by the DMN are still largely unknown. Previous evidence suggested that the activity of the DMN was bound to be related to alpha (8–13 Hz) and beta (14–30 Hz) oscillations (Knyazev, Slobodskoj‐Plusnin, Bocharov, & Pylkova, 2011; Mantini, Perrucci, Del Gratta, Romani, & Corbetta, 2007).

Studies have found altered functional connectivity (FC) within the DMN in several types of epilepsy, including idiopathic generalized epilepsy (Gotman et al., 2005; Kay et al., 2013), temporal lobe epilepsy (Haneef, Lenartowicz, Yeh, Engel Jr, & Stern, 2014; Liao et al., 2011; Robinson et al., 2017), absence epilepsy (Luo et al., 2011), benign epilepsy with centrotemporal spikes (Li et al., 2019), and infantile spasms (Wang, Li, Wang, Chen, & Huang, 2017). However, no study has specifically focused on the alteration of FC in the DMN in patients with cingulate gyrus epilepsy. For the first time, we used magnetoencephalography (MEG) to study the electrophysiological correlates of the DMN in cingulate gyrus epilepsy. Because the PCC is considered the only node in the DMN to directly interact with almost all other nodes, it may yield new information about the DMN and suggest a potential cingulate gyrus epilepsy biomarker.

## 2 METHODS

### 2.1 Subjects

Thirty subjects, comprising of 15 patients with cingulate gyrus epilepsy and 15 healthy controls, underwent resting‐state MEG scanning. We reviewed and analyzed the data of 15 surgically treated cases of lesional cingulate gyrus epilepsy that were seizure‐free or had markedly reduced seizure frequency. Cases underwent resective epilepsy surgery between January 2010 and May 2017 at Xuanwu Hospital Capital Medical University and the Beijing Epilepsy Center. The epileptogenic zone was verified by clinical symptoms, magnetic resonance imaging (MRI), video electroencephalogram (VEEG), intraoperative electrocorticography, neuropsychological evaluation, and good postsurgical reduction in seizure frequency. The history, clinical manifestations, radiological findings, neurophysiological data, and surgical outcomes of participants were reviewed. Resting‐state MEG scanning was carried out on patients at the time of hospitalization. Fifteen healthy controls, recruited from the general population via advertisements and statistically matched to the 15 patients for age, gender, and education, were used for comparison. The demographic characteristics of the subjects are summarized in Table 1.

**Table 1:** Demographic and clinical characteristics of the subjects

	Epilepsy group	Healthy control group
Sample size	15	15
Gender (male/female)	11/4	9/6
Age (years)	21.2 ± 4.9	24.4 ± 5.1
Education (years)	11.8 ± 4.2	14.9 ± 3.2
Handedness (right/left)	15/0	15/0
Duration of illness (years)	12.1 ± 5.2	–

*Note*: Data are presented as counts and mean ± standard deviation.

The inclusion criteria for the patients with cingulate gyrus epilepsy were as follows: (a) surgical resection that included parts of the cingulate gyrus followed by a seizure‐free outcome or a marked reduction in seizure frequency; (b) more than 12 months of postsurgical follow‐up; and (c) right‐handedness. Exclusion criteria were as follows: (a) presence of a metal implant, such as a vagus nerve stimulation (VNS) device or metallic denture; (b) clinically significant systemic organic disease; (c) history of another major neurological or psychiatric disease; and (d) inability to keep still during MEG recordings or MRI scans.

This study was approved by the Medical Ethics Review Committee of Xuanwu Hospital Capital Medical University. Neurologists were responsible for the selection and exclusion of subjects, and written informed consent was obtained from all subjects.

### 2.2 MEG recordings

MEG data were acquired in a magnetically shielded room with a whole‐head MEG system with 306 channels (VectorView™, ElektaNeuromag, Helsinki, Finland) at the MEG Center at Xuanwu Hospital Capital Medical University.

Before recording data, three coils were fixed to the left and right pre‐auricular points and nasion of each subject. A head localization procedure was implemented before and after each acquisition to locate the patient's head relative to the coordinate system fixed to the MEG system. Subjects were put in a supine position with their eyes slightly closed and requested to remain still. Head movement was limited to 5 mm during each recording. If a patient's head moved more than 5 mm, the data were excluded and a new dataset was recorded for the subject. All MEG data were recorded at a sampling rate of 1,000 Hz. In addition, empty room MEG recordings were routinely performed to monitor system and environmental noise.

### 2.3 MRI scan

Three‐dimensional MRI was acquired using a 3‐T scanner (Siemens Magneton Vision; Siemens, Munich/Erlangen, Germany). Three fiduciary marks were laid on identical locations to the positions of the three coils used in the MEG recordings with the aid of digital photographs to achieve an accurate co‐registration of the two data sets. Subsequently, all anatomic landmarks were made visible in the MRI scans.

### 2.4 Preprocessing and source reconstruction

The MEG data were preprocessed with the Elekta Maxfilter using a band‐stop filter to remove power‐line interference (50 Hz). The deviated trials and channels were removed. Then, the MEG data from the resting state condition were extracted and fragmented into consecutive 500‐ms epochs using MEG Processor software (https://sites.google.com/site/braincloudx/). To remove interference caused by interictal spikes in cingulate gyrus epilepsy, the epochs of MEG data were visually examined. For MEG spike detection in each epoch, superimposed MEG signals from 306 channels were screened. Sharp signals that were easily identifiable from ongoing background activity were rejected and regarded as probable MEG spikes. Any epoch with a probable MEG spike was abandoned from the present study. In addition to spike elimination, we excluded epochs with sharp artifact signals due to clear contributions from heartbeats, eye movements, or other physiological signals. For each subject, 40 epochs without spikes or artifacts were randomly selected for further analysis.

Previous evidence has suggested that the activity of the DMN is related to alpha (8–13 Hz) and beta (14–30 Hz) oscillations (Knyazev et al., 2011; Mantini et al., 2007). In this study, we chose alpha (8–13 Hz), beta (14–30 Hz), and gamma (31–80 Hz) band oscillations to reconstruct sources with resting‐state MEG data.

To study the neuromagnetic network at source level, we localized significant neuromagnetic activities using accumulated source imaging (Babiloni et al., 2005; Xiang et al., 2014, 2015) that was defined as the volumetric summation of source activity over a period of time. Accumulated source imaging was based on the following equation:

(1) $$\mathrm{Asi}\left(r,s\right)=\sum_{t=1}^{t=n}Q\left(r,t\right)$$

In Equation (1), Asi delegates accumulated source strength at location *r*; *s* represents the time slice; *t* stands for the time point of MEG data; *n* indicates total time points of MEG data, and *Q* indicates the source activity at source *r* and at time point *t*. We defined that *s* ≥ 1 and *s* ≤ *n*/2. We applied two‐step beamforming to compute the source activity (Barnes, Hillebrand, Fawcett, & Singh, 2004; Sekihara, Nagarajan, Poeppel, Marantz, & Miyashita, 2001; Xiang, Tenney, et al., 2015). The first step calculated lead fields for each source. The second step produced matrices with MEG data. In the third step, we chose sensors for partial sensor coverage for each voxel with lead field (Xiang et al., 2015), defined as voxel‐based partial sensors. Next, we computed the covariance of voxel‐based partial sensors. Then, we computed two sets of magnetic source images through a vector beamformer (Xiang, Korman, et al., 2015). The next step estimated a coherent source and source orientation using the covariance matrix‐vector beamformer. When the source direction was worked out, the last step generated the source activity with the scalar beamformer (Xiang, Korman, et al., 2015). The detailed mathematical algorithms were described in previous reports (Xiang et al., 2014; Xiang, Tenney, et al., 2015). In our study, the whole brain was scanned at a 6‐mm resolution (around 17,160 sources). When the distance between two voxels was shorter than 10 mm, they were regarded as one source.

### 2.5 Dynamic functional connectivity estimation

Based on previous research (Dai, Zhang, Dickens, & He, 2012; Xiang et al., 2014; Xiang, Korman, et al., 2015), functional networks were analyzed at the source level. In this study, whole‐brain source neural networks were estimated by analyzing the correlation of all voxels signals through the aforementioned algorithms. In particular, the correlation of signals from a two source pair was statistically analyzed using the computing correlation coefficient. The correlation factors were based on the following equation:

(2) $$R\left(X_{a},X_{b}\right)=\frac{C\left(X_{a},X_{b}\right)}{SX_{a}SX_{b}}$$

where *R*(*X*_a_,*X*_b_) represents the correlation of a source pair in two locations (“a” and “b”). The *X*_a_ and *X*_b_ represent signals in each of the two sources, which were paired for calculating connection. *C* (*X*_a_, *X*_b_) indicates the mean of the signals in the two sources. *SX*_a_ and *SX*_b_ indicate the standard deviation of the signals from the two sources. Moreover, every possible connection was analyzed for each dual‐source pair at the source level to reduce possible variance.

The same data analyses were also applied to the MEG data obtained from the 15 healthy subjects. MEG Processor software (Cincinnati, OH) was used to perform the aforementioned calculation.

### 2.6 Degree of node estimation

In this study, we also estimated the node degree using graph theory. In graph theory, the degree si of a node *i* was the number of edges linking to the node and was defined as:

(3) $$\mathrm{si}=\sum_{j}w_{\mathrm{ij}}$$

where *w*_ij_ represented the edge that connected node i and node j. The node degree, si, can be used to determine the extent to which the node was central in the graph. We think that investigating both the connection strength and the node degree may reveal different information about functional integrations of the brain network. The above calculation was performed using MEG Processor software (Cincinnati, OH).

### 2.7 Regions of interest for the DMN

Different DMN coordinates were suggested in previous resting‐state fMRI studies (Grady, Luk, Craik, & Bialystok, 2015; Sasai et al., 2014). Common regions of interest (ROIs) were defined in the DMN, and similar coordinates for major nodes were provided. Considering the different spatial resolutions of fMRI and MEG, in this study ROI coordinates were adapted from de Pasquale et al. because of their continuous application of MEG (de Pasquale et al., 2012; Schafer, Morgan, Ye, Taylor, & Doesburg, 2014). In this study, 12 seed ROIs were defined for the DMN with 6 mm radii spheres: The left angular gyrus (LAG), right angular gyrus (RAG), left posterior cingulate/precuneus cortex (LPCC), right posterior cingulate/precuneus cortex (RPCC), left inferior temporal gyrus (LITG), right inferior temporal gyrus (RITG), left ventral medial prefrontal cortex (LvMPFC), right ventral medial prefrontal cortex (RvMPFC), left dorsal medial prefrontal cortex (LdMPFC), right dorsal medial prefrontal cortex (RdMPFC), left anterior cingulate cortex (LACC), and right anterior cingulate cortex (RACC). More information about the ROIs is summarized in Table 2. According to a previous study on the spatial partition of the DMN (Wei et al., 2015), the LvMPFC, RvMPFC, LdMPFC, RdMPFC, LACC, and RACC comprise the anterior DMN, and the LAG, RAG, LPCC, RPCC, LITG, and RITG comprise the posterior DMN.

**Table 2:** Regions of interest (ROIs) for DMN and MNI coordinates

Common names	Abbreviation	MNI coordinates
Left angular Gyrus	LAG	(−43, −76, 35)
Right angular Gyrus	RAG	(51, −64, 32)
Left posterior cingulate/Precuneus cortex	LPCC	(−3, −54, 31)
Right posterior cingulate/Precuneus cortex	RPCC	(3, −54, 31)
Left ventral medial prefrontal cortex	LvMPFC	(−2, 51, 2)
Left dorsal medial prefrontal cortex	LdMPFC	(−13, 52, 23)
Right dorsal medial prefrontal cortex	RdMPFC	(2, 53, 24)
Right ventral medial prefrontal cortex	RvMPFC	(2, 51, 2)
Left inferior temporal Gyrus	LITG	(−57, −25, −17)
Right inferior temporal Gyrus	RITG	(57, −25, −17)
Left anterior cingulate cortex	LACC	(−6, −36, 31)
Right anterior cingulate cortex	RACC	(6, −36, 31)

*Note*: ROI coordinates were adapted from de Pasquale et al. (2012).

### 2.8 Statistical analysis

An independent sample *t*‐test was performed to examine significant differences between groups (patients with cingulate gyrus epilepsy versus control subjects) in network parameters (node degree and strength). The correlation between the clinical characteristics of the patients (age, gender, duration of epilepsy, and seizure frequency) and the strength of each FC was analyzed using Spearman's correlation coefficients. Statistical analyses were performed using SPSS version 23.0 (SPSS Inc., Chicago, IL). All tests were two‐sided, and the threshold of statistical significance for differences was *p* < .05 for each test.

## 3 RESULTS

### 3.1 Clinical characteristics of patients with cingulate gyrus epilepsy

Fifteen patients met inclusion criteria, including 11 men and 4 women, with a mean age of 21.2 ± 4.9 years and a mean epilepsy duration of 12.1 ± 5.2 years. None had a family history of seizures. The neurological examination was normal in all patients. One patient had undergone previous surgical resection of the temporal lobe and hippocampus, and one patient had undergone previous surgical resection of the frontal lobe; neither had benefited with respect to seizures. Intraoperative electrocorticography (ECoG) was used to identify epileptogenic areas and guide epilepsy surgery in all patients.

Twelve patients (80%) were seizure‐free at >12 months follow‐up. Three patients (20%) were almost seizure‐free. Gender, age, epilepsy duration, and MRI (positive or negative) were not significantly associated with a seizure‐free outcome. Pathological abnormalities were found in all patients; 14 patients (93%) had focal cortical dysplasia (FCD; 7 ILAE Type I, 5 Type II, and 2 ILAE Type III); one had malformation of cortical development. The existence of FCD in the surgical specimen was not associated with a seizure‐free outcome. The detailed clinical characteristics of the patients are summarized in Table 3.

**Table 3:** Clinical characteristics of patients with cingulate gyrus epilepsy

#	Gender/age (year)	Epilepsy onset (age, y)	Epilepsy duration (year)	Seizure frequency (times/day/month)	Aura	Ictal seminologic findings	Seizure duration	Scalp VEEG		MEG (dipole)	ICEEG			Resection		Histopathology	MRI	Outcome
								Interictal EEG	Ictal EEG		Intericta1	Ictal	Intraoperative ECoG	Side	Site			
1	F/23	6	17	10–30/day	Fear	Ictal yell, automotor seizure, hypermotor seizure	Seconds	Bifrontal spike‐slow waves	Bilateral frontotemporal fast waves	Left anterior F	Bi ACC, Lt basal part of F, Lt anterior I, Lt SMA	Lt ACC, Lt anterior I, Lt SMA	Lt ACC, Lt SFG, Lt basal part of F	Lt	ACC, SFG & Fp	FCDIIb	Negative	Sz free (follow‐up: 39 months)
2	M/23	19	4	1–2/month	–	Automotor seizure, head turn right, tonic clonic seizure	Minutes	Left frontal spike‐slow waves	Left frontal fast activities	Left posterior F	Lt ACC, Lt Fp, Lt SFG	Lt SFG, Lt ACC, Lt Fp	Lt ACC, Lt SFG	Lt	ACC & SFG	FCDI	Negative	Sz free (follow‐up: 32 months)
3	M/21	16	5	30–40/day	Headache	Automotor seizure, shout, hypermotor seizure	Seconds	Right frontal sharp and slow waves	Right frontal fast activities	Right F, T and left F, P	Bi ACC, Rt F, Bi H	Rt ACC, Rt F,	Rt ACC, Rt SFG & Fp	Rt	ACC, SFG & Fp	FCDIIa	Negative	Sz free (follow‐up: 46 months)
4	F/23	3	20	1–4/day	Fear, fantasy	Shout, fear, hypermotor seizure	Seconds	Bioccipital sharp slow waves, bilateral sphenoid sharp waves	Whole brain low amplitude fast waves	Left mid F	Lt CC, Lt basal part of F, Lt C	Lt CC, Lt basal part of F	Lt CC, Lt C, Lt I	Lt	ACC & Fp	FCDI	Negative	Sz free (follow‐up: 39 months)
5	F/24	7	17	15–20/month	Fear, blurred vision	Gelastic seizure, vocalization, spin around, automotor seizure, stare blankly, red‐faced	Seconds	Bilateral frontotemporal spike, sharp and slow waves	Right frontotemporal fast activities	Right O	Rt ACC, Rt F	Rt ACC, Rt F	Rt ACC, Rt SFG	Rt	ACC, SFG & Fp	FCDIIa	Negative	Sz free (follow‐up: 72 months)
6	M/27	10	17	1–4/day	–	Shout, automotor seizure, head turn right, clonic seizure	Minutes	Left frontotemporal and central sharp and slow waves, low amplitude fast waves	Bilateral central low amplitude fast waves	Bilateral T	Bi F, Bi C	Lt SFG	Lt MCC, Lt basal part of F	Lt	MCC, MFG & IFG	FCDI	Negative	Sz free (follow‐up: 12 months)
7	M/14	6	8	3–4/day	Fear	Spin around, vocalization, hypermotor seizure	Seconds	Left temporal sharp waves, left frontotemporal slow wave	Left frontal low amplitude fast activities	The lower left F	Lt ACC, & MCC, Lt F, Lt I	Lt ACC, & MCC, Lt F	Lt MCC, Lt I	Lt	ACC, MCC, MFG & IFG	FCDIIb	Postoperative changes of left frontal	Sz free (follow‐up: 36 months)
8	M/18	10	8	2–4/day	Panic, irritability, sexual excitement	Hypermotor seizure, vocalization, left mouth twitch, automotor seizure, tonic clonic seizure	Seconds	Right frontotemporal sharp waves, low amplitude fast waves	Right frontotemporal low amplitude fast activities	Left T	Rt basal part of F, Rt basal part of T, Rt I	Rt CC, Rt basal part of F, Rt basal part of T, F operculum	Rt CC, Rt basal part of F, Rt basal part of T	Rt	CC, basal part of F, Fp & operculum	FCDIIId	Right anterior temporal hippocampus after resection	Sz free (follow‐up: 60 months)
9	M/26	8	18	20–40/days	Dizziness	Automotor seizure, head turn left, left mouth twitch	Seconds	Right frontal spike, right parietal sharp waves	Right frontal and right parietal fast activities	Right P and anterior upper F	Rt F, Rt P	Rt P	Rt PCC, Rt F, Rt P	Rt	PCC & superior P	FCDI	Negative	Sz free (follow‐up: 38 months)
10	M/22	5	17	20–40/day	Fear	Shout, fear, clonic seizure, red‐faced, automotor seizure	Seconds	Bilateral frontal spike‐slow waves	Bilateral frontal low amplitude fast activities	Right frontal longitudinal fissure	Bi F	Bi F	Lt CC, Lt F	Lt	CC, MFG & SFG	MCD	Negative	Sz free (follow‐up: 84 months)
11	M/10	1	9	2–3/day	–	Head eye left oblique, left limb stiffness, red‐faced	Seconds	Right central, parietal, temporal sharp and slow wave; left frontal, temporal slow wave	Bilateral frontal, central sharp waves	Right middle and posterior F	–	–	Lt MCC, PCC, P& F	Lt	MCC, PCC, P& SFG	FCDIa	Negative	90% sz reduction (follow‐up: 23 months)
12	M/22	13	9	3–6/day	Panic, irritability	Head eye right oblique, tonic clonic seizure	Minutes	Right frontal sharp and slow waves	Left leadsθrhythm, right leads spike‐slow waves, multi‐spikes and slow waves	Bilateral posterior F	–	–	Lt MCC, MFG & SFG	Lt	MCC, MFG & SFG	FCDIIId	Negative	Sz free (follow‐up: 68 months)
13	F/30	15	15	2–3/day	–	Vocalization, hypermotor seizure, automotor seizure	Minutes	Bilateral frontotemporal sharp and slow waves	Bilateral frontal low amplitude fast activities, right frontotemporal sphenoid slow waves	Right middle and lateral T	Rt ACC, Rt anterior T, Rt anterior I	Rt ACC, Rt anterior T, Rt anterior I	Rt ACC, Rt anterior T, Rt anterior I	Rt	ACC, basal part of F & anterior T	FCDI	Negative	90% sz reduction (follow‐up: 39 months)
14	M/17	11	6	2–20/day	Fear, panic	Vocalization, hypermotor seizure, automotor seizure, tonic seizure	Seconds	Bilateral frontal sharp waves	Bilateral frontal sharp and slow waves, right significant	Right anterior and lower T	Rt CC, Rt I	Rt ACC, Rt I	Rt CC, Rt I, Rt F	Rt	ACC, IFG & Fp	FCDI	Negative	90% sz reduction (follow‐up: 39 months)
15	M/18	7	11	1–2/day	Intracerebral overinductance	Tonic seizure, hypermotor seizure, automotor seizure, head left oblique, tonic clonic seizure	Seconds	Bilateral frontal slow waves, right sphenoid sharp waves	Right sphenoid sharp waves	Right posterior ITG	Bi ACC, Bi F, Bi SMA	Rt C, Rt F, Rt SMA	Rt ACC, Rt F, Rt T, Rt SMA	Rt	ACC, MCC & SFG	FCDIIb	Negative	Sz free (follow‐up: 16 months)

Abbreviations: ACC, anterior cingulate cortex; C, central; CC, cingulated cortex; F, frontal; FCD, focal cortical dysplasia; Fp, frontopolar; I, insula; IFG, inferior frontal gyrus; ITG, inferior temporal gyrus; Lt, left; MCC, middle cingulate cortex; MFG, middle frontal gyrus; O, occipital; P, parietal; PCC, posterior cingulate cortex; Rt, right; SFG, superior frontal gyrus; SMA, supplementary motor area; T, temporal.

### 3.2 FC analysis of the DMN connectivity between patients with cingulate gyrus epilepsy and controls

**Figure 1:**
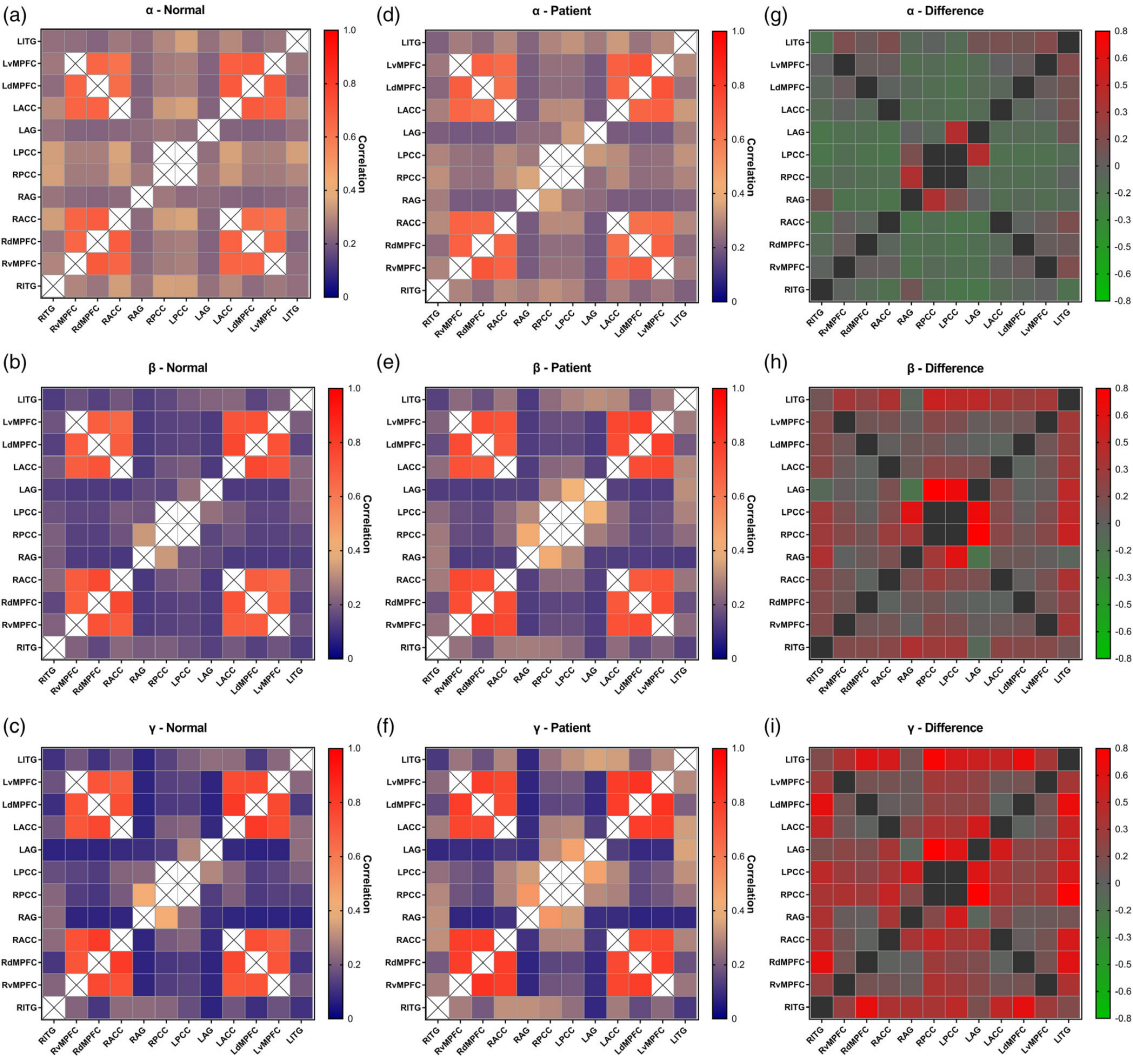
Matrices of the mean functional connectivity (FC) strength of the default mode network (DMN) regions of interest for α, β, and γ bands. Matrices shown separately for the control group (Normal) and cingulate gyrus epilepsy group (Patient), and the difference in DMN FC strength between the two groups

In the FC analysis, we found that the FC within the DMN was significantly greater than 0 in the control group and patient group. The mean DMN FC strengths of the two groups and the difference of DMN FC strength between the two groups are shown in Figure 1. As shown in Figure 1, similar FC strength was shown in homologous bilateral brain regions. The higher the frequency was, the greater and more common were differences of FC between the two groups.

**Figure 2:**
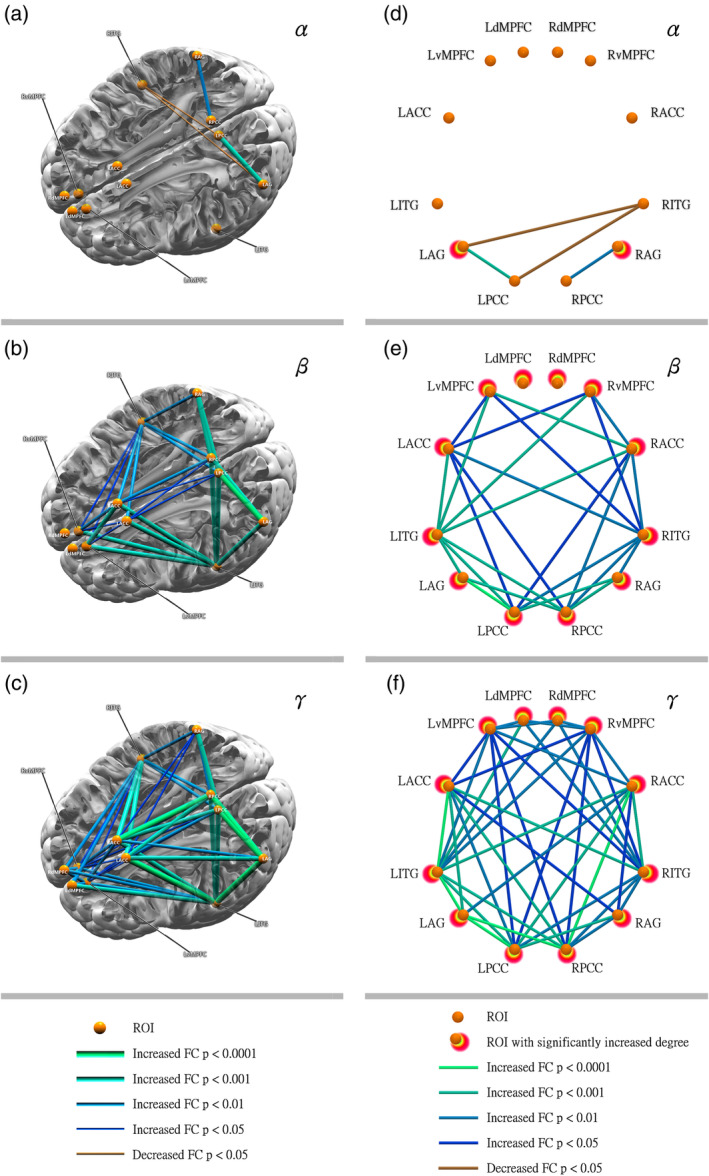
(a–c) Significantly different functional connectivity (FC) within the default mode network (DMN) in a pseudoanatomical organization between the control group and cingulate gyrus epilepsy group. (d–f) Regional connectivity pairs with a significant between‐group difference

The between‐group differences in the DMN, which was defined by the strength of FC with seeds in the DMN, were analyzed by an independent sample *t*‐test. The FC strength in the patients with cingulate gyrus epilepsy was lower than that of the healthy control subjects in only two pairs (LPCC–RITG and LAG–RITG) in the alpha band. However, significantly (*p* < .05) higher FC strength was found for all three frequency bands (alpha (8–13 Hz), beta (14–30 Hz), and gamma (31–80 Hz) band), as shown in Figure 2. In the alpha band, only two FC strengths (LAG–LPCC and RAG–RPCC) were significantly higher in the cingulate gyrus epilepsy group. In the beta band, significantly higher FC in the patient group was observed in more locations than in the alpha band (26 locations had significantly higher FC). In the gamma band, the patients had significantly higher FC more frequently than controls (42 FC strengths were significantly higher). The details of the significantly higher FCs of the patients are shown in Figure 2. The number of significantly higher FC strengths was equal in bilateral hemispheres. Notably, as shown in Figure 2, the effect of cingulate gyrus epilepsy upon DMN connectivity on the left side was more obvious than that on the right side.

### 3.3 Node degree analysis between patients with cingulate gyrus epilepsy and controls

Not surprisingly, in the node degree analysis, the results were similar to those of the FC analysis. We found that the difference in node degree between the control group and cingulate gyrus epilepsy group was most obvious when the correlation factor was >0.3. With increasing MEG frequency, the difference in node degree between the two groups became significantly greater.

**Figure 3:**
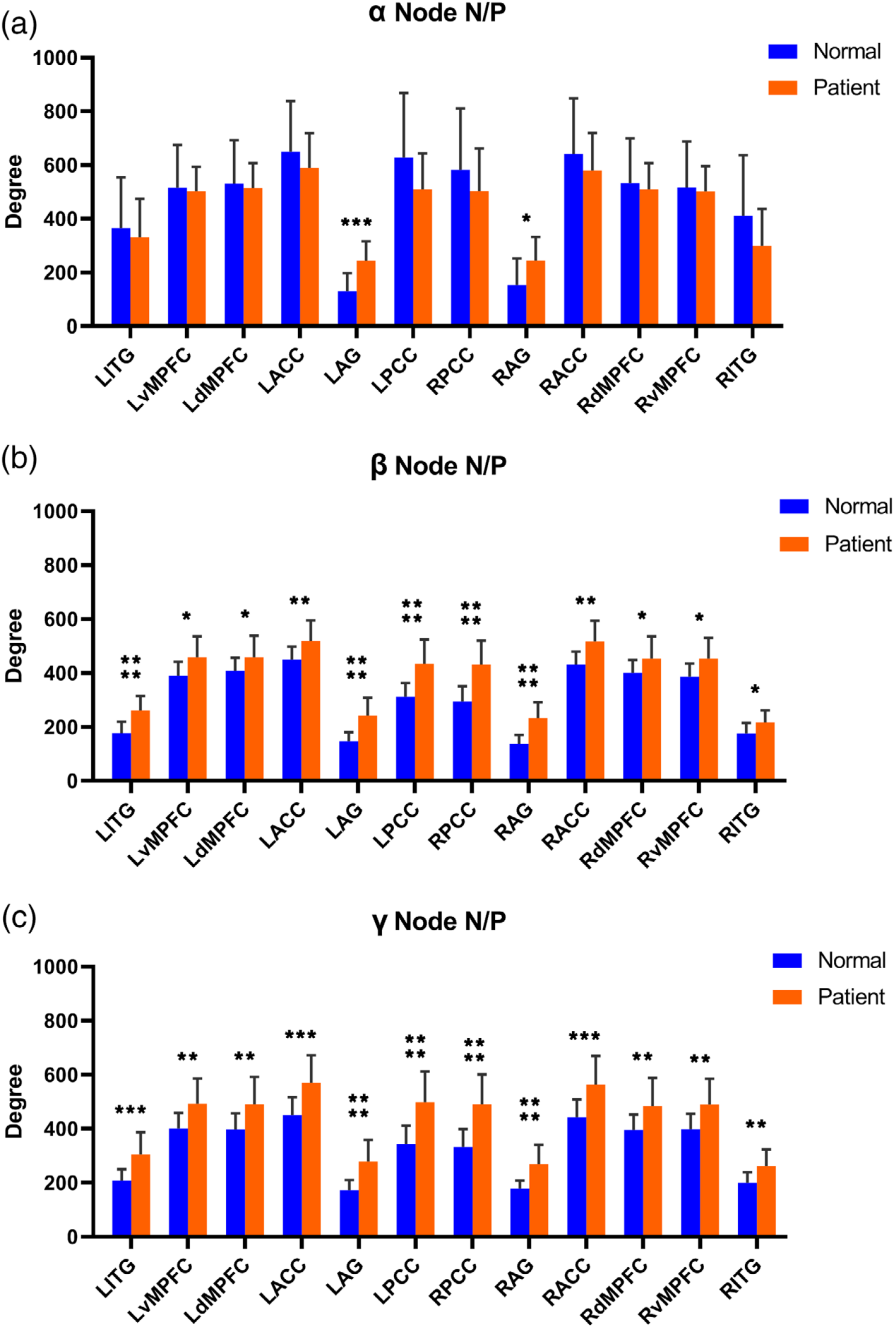
Bar graph comparing the node degree for each region of interest (ROI) within the default mode network (DMN) of the two study groups. The symbols *, **, ***, and **** denote a significant difference of *p* < .05 corrected, *p* < .01, *p* < .001, and *p* < .0001, respectively, between the cingulate gyrus epilepsy group (Patient) and control group (Normal)

Compared with healthy control subjects, no nodes had a significantly lower node degree in patients with cingulate gyrus epilepsy. However, in all frequency bands, nodes with a significantly higher node degree were found (*p* < .05), as shown in Figure 3. In the alpha band, only two nodes (LAG and RAG) had a significantly higher node degree in the cingulate gyrus epilepsy group. Other nodes had a lower degree, but there was no statistically significant difference (*p* < .05). In the beta band, all selected ROIs had a significantly higher node degree. Likewise, in the gamma band, all selected ROIs had a significantly higher node degree, but the differences were greater than in the beta band. As shown in Figure 3, the bilateral AG showed the most significantly higher node degree in all selected bands.

### 3.4 γ band neural activity may play a key role in DMN connectivity

Surprisingly, we found a special phenomenon in this study. The significantly higher FC between the cingulate gyrus epilepsy and control groups became more marked with greater MEG frequency (shown in Figure 2). In the alpha band, only two FCs were significantly higher in the patient group. This increased to 26 in the beta band. The gamma‐band had 42 significantly higher FCs in the patient group. Moreover, for the same FC, the difference between study groups was greater with higher frequency. Similar to the FC, with higher frequency, the difference in node degree between the two groups was increasingly more significant (shown in Figure 3). In the alpha band, only two nodes showed a significantly greater node degree in the cingulate gyrus epilepsy group. In the beta band, all 12 selected ROIs had a significantly higher node degree. In the gamma band, all selected ROIs also had a significantly higher node degree, but the difference was greater than for the beta band. Therefore, the most significant differences between cingulate gyrus epilepsy and controls were observed in the γ band. Thus, we consider that γ band neural activity may be a key contributor to MEG‐based DMN connectivity.

### 3.5 Spatially specific FC between the LAG and LPCC in the seed‐based maps

**Figure 4:**
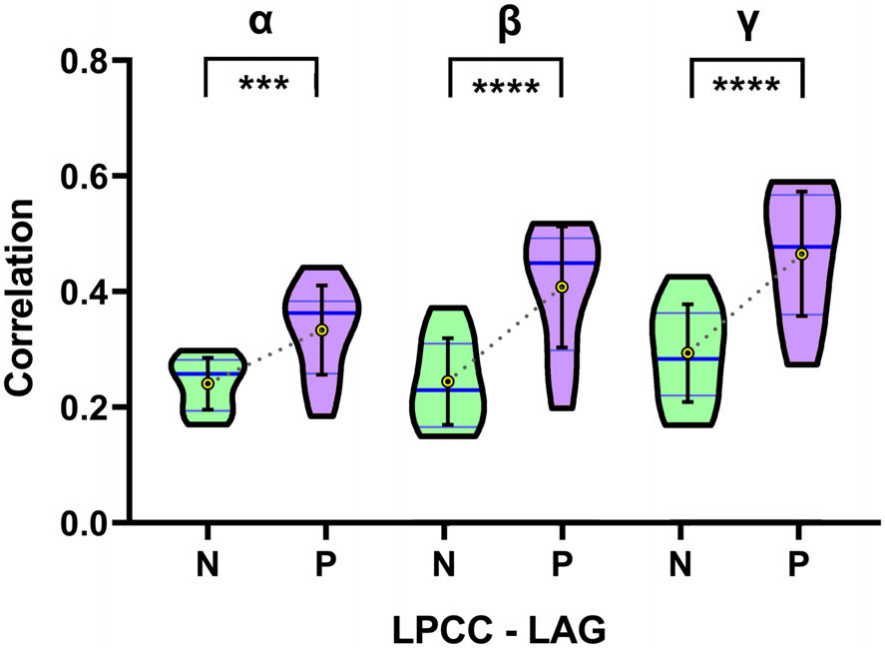
Comparison of the functional connectivity (FC) strength for the left posterior cingulate cortex‐left angular gyrus (LPCC–LAG) pair between the control group (normal) and cingulate gyrus epilepsy group (patient) in violin plots. N, normal; P, patient. *** denotes a *p* < .001 significant difference and **** a *p* < .0001 significant difference

In the present study, we found that one FC pair (LPCC–LAG) had the most significant differences between the cingulate gyrus epilepsy and control groups in all three frequency bands. In the alpha band, only two significantly higher FCs (LAG–LPCC and RAG–RPCC) were found. The LAG–LPCC FC was significantly higher than the RAG–RPCC FC. In the beta and gamma bands, the significantly higher FCs were greater than in the alpha band. However, the most remarkable difference was located in the FC between the LAG and LPCC. Moreover, the between‐group difference was increasingly significant with greater frequency: the γ band had the most significant difference (shown in Figure 4). Consequently, we considered that the strength of the FC between the LAG and LPCC was significantly associated with cingulate gyrus epilepsy. It may be useful as a biomarker for cingulate gyrus epilepsy.

### 3.6 Correlation analyses

For each ROI, FC values that showed significant between‐group differences were extracted. Subsequently, the correlation analysis was performed. No significant correlations were observed between FC and lesion side, epilepsy duration, gender, or age of onset of epilepsy.

## 4 DISCUSSION

### 4.1 Cingulate gyrus epilepsy is difficult to diagnose

In 1989, cingulate gyrus epilepsy was classified as a form of frontal lobe epilepsy (FLE) by the ILAE (Commission on Classification and Terminology of the International League Against Epilepsy., 1989). FLE is considered as the second most common type of epilepsy, but detecting seizure onset in FLE is laborious. Previous clinical studies showed that FLE represents an important cause of refractory epilepsy. Therefore, the accurate diagnosis of FLE and earlier treatment are essential.

The cingulate gyrus is located in the medial pericallosal aspect of each frontal lobe. Distinguishing cingulate gyrus epilepsy from other FLE syndromes based on semiological results alone is difficult. Furthermore, the cingulate gyrus is not readily accessible for routine scalp EEG, and its close proximity between the right and left cingulate gyrus also increases the difficulty in identifying where seizures actually initiate. Therefore, in nonlesional cases, differentiation without intracranial ictal recordings is difficult, leading to finite corticectomy and excellent seizure outcome. Consequently, an appropriate biological marker for cingulate gyrus epilepsy is urgently needed. In this study, we assessed 15 patients with cingulate gyrus epilepsy retrospectively. We summarized the detailed clinical and electrophysiological characteristics of the patients to help clinicians to further understand cingulate gyrus epilepsy.

### 4.2 Increased FC between the LAG and LPCC may be useful as a biomarker for cingulate gyrus epilepsy

Since the symptoms and electrophysiological examinations of cingulate gyrus epilepsy lack specificity, we explored alternative diagnostic markers. The PCC is known to be one of the core nodes in the DMN, and the PCC is considered as the only node in the DMN that directly interacts with almost all other nodes according to Fransson and Marrelec (2008). Therefore, epilepsy in the cingulate gyrus may lead to characteristic changes of FC in the DMN. Surprisingly, we found a particular FC pair (LAG–LPCC) that showed the greatest significant differences between patients with cingulate gyrus epilepsy and controls in all three frequency bands (α, β, and γ) studied. Moreover, the differences were increasingly more significant with higher frequency; the γ band had the most significant between‐group difference. Thus, we propose that high FC between the LAG and left PCC may be used as a biomarker for cingulate gyrus epilepsy.

The cingulate gyrus and AG have close fibrous connections. In addition, they are both functionally related to cognition and sleep (Ding et al., 2018). In the DMN, FC between the AG and PCC is relatively stronger than others (de Pasquale et al., 2012). Therefore, it is reasonable that cingulate gyrus epilepsy leads to significantly increased FC between the LAG and left PCC. Nevertheless, it is surprising that the difference is so obvious. Perhaps some other relationships between the LAG and left PCC merit further investigations.

### 4.3 Chronic cingulate gyrus epilepsy impairs intrinsic brain activity of the DMN and causes functional reorganization and plasticity of the DMN

Previous studies have reported a reduction in FC in the DMN in patients with mesial temporal lobe epilepsy (Liao et al., 2011). Wang et al. (2010) previously estimated the resting‐state networks in patients with generalized tonic–clonic seizures and found regions with decreased as well as increased FC in the DMN. The basal FC in children with multifocal epilepsy was evaluated by Siniatchkin et al. (2018), and they found a trend towards increased connectivity in the DMN. Li et al. (2019) reported an increase in FC in the DMN in children with benign epilepsy with centrotemporal spikes. Wang et al. (2017) evaluated the resting‐state connectivity of children with infantile spasms and found reduced connectivity in the DMN. In our study, we found only two lower FCs (LPCC‐RITG and LAG–RITG) in the alpha band in patients with cingulate gyrus epilepsy compared to controls. However, significantly higher FC was found in all selected frequency bands. Different types of epilepsy have distinct effects on the DMN. Chronic epilepsy generally impairs cognition, but it might also contribute to functional reorganization and plasticity (Elger, Helmstaedter, & Kurthen, 2004). A previous study suggested that the functional integration of the DMN could be comparatively weak during childhood, and then become gradually stronger during development (Fair et al., 2008). Therefore, early epileptic seizures in patients with epilepsy may disturb the development of the DMN and change the structure of the DMN. Likewise, chronic cingulate gyrus epilepsy impairs intrinsic brain activity of the DMN and causes functional reorganization and plasticity of the DMN. Further research is needed to determine the clinical relevance of this abnormal FC.

### 4.4 Activity of the DMN is likely related to γ band neural activity

In previous studies, research on FC in the DMN has uncovered a wide spectrum of oscillatory components, among which, the alpha and beta frequency bands are the most frequently reported (Brookes et al., 2011; de Pasquale et al., 2010, 2012; Foster et al., 2016; Hipp, Hawellek, Corbetta, Siegel, & Engel, 2012; Jerbi et al., 2010; Mantini et al., 2007; Marzetti et al., 2013). One study suggested a positive correlation between fluctuations of the blood‐oxygen‐level‐dependent (BOLD) signals in the DMN and alpha power fluctuations (Mantini et al., 2007). Recent MEG evidence suggested that the DMN was a hub of inter‐network cortical interactions in the resting state, especially in the alpha and beta frequency bands (de Pasquale et al., 2012). However, in our study, we found that the most significant differences between cingulate gyrus epilepsy and controls in terms of FC in the DMN were in the γ band. As the cingulate gyrus is one of the core nodes in the DMN, and the PCC is considered as the only node in the DMN that directly interacts with almost all other nodes, we suggest that FC in the DMN was likely related to γ band oscillations (31–80 Hz).

### 4.5 MEG is useful for investigating intrinsic brain activity

MEG, a well‐established technology with better temporal resolution than functional MRI, has shown the ability to contribute to investigations of intrinsic brain activity. In our study, we investigated the DMN in patients with cingulate gyrus epilepsy at the source level, whereas many previous studies have analyzed the DMN at the sensor or electrode level. The entire brain was analyzed in this study, which may make the findings more objective than those using a few contacts.

## 5 CONCLUSION

Our findings identify specific components of the DMN that are affected in cingulate gyrus epilepsy. The results of this study suggest that γ band neural activity may be a key contributor to MEG‐based DMN connectivity. Furthermore, the high FC between the LAG and left PCC may be useful as a biomarker for cingulate gyrus epilepsy.

## CONFLICT OF INTEREST

The authors report no competing interests.

## Data Availability

Data availability The authors confirm that the data supporting the findings of this study are available within the article.
